# Dental Implant Survival Rates: Comprehensive Insights from a Large-Scale Electronic Dental Registry

**DOI:** 10.3390/jfb16020060

**Published:** 2025-02-11

**Authors:** Guy Tobias, Tali Chackartchi, Doron Haim, Jonathan Mann, Mordechai Findler

**Affiliations:** 1 Department of Community Dentistry, Hebrew University-Hadassah School of Dental Medicine, Jerusalem 76841, Israel; mann.hadassah@gmail.com; 2 Department of Periodontology, Hebrew University-Hadassah School of Dental Medicine, Jerusalem 91120, Israel; tali222@hotmail.com; 3 Research Unit Maccabi-Dent, Tel Aviv 6801298, Israel; haim_d@maccabi-dent.com (D.H.); findlermo@gmail.com (M.F.); 4 Oral Medicine Unit, Sheba Medical Centre, Tel-Hashomer 5262000, Israel

**Keywords:** dental implant, early failure, osseointegration, risk factors

## Abstract

Background: This descriptive study aimed to assess the survival rates and outcomes of dental implants in one of the four national HMOs in Israel. Data are provided for the period from 1 January 2014 to 31 December 2022. Materials and Methods: This retrospective analysis utilized electronic medical records of patients who underwent dental implant placement during the specified period. Statistical analyses included chi-squared tests, Student’s *t*-tests, and generalized estimating equation (GEE) analyses to identify potential risk factors associated with early and late implant failures. Results: A total of 158,824 dental implants were placed in 53,874 patients. The overall implant failure rate was 2.21%, while the early failure rate during the osseointegration phase—before prosthetic reconstruction—was 1.56%. Significant associations with implant failure were observed for male patients (2.53% failure rate), implants in the maxillary molar region (3%), and the central incisor region (3.37%), approximately double the failure rates seen in other implant sites (*p* < 0.001). Conclusions: This extensive data analysis demonstrates a low overall failure rate for dental implants. The highest failure incidence occurred within the first year post-implantation, declining in subsequent years irrespective of rehabilitation status. Early failure risk factors differ based on various factors and should be carefully integrated into presurgical planning.

## 1. Introduction

Dental implants are a well-established treatment option for replacing missing teeth, showing high survival rates when placed in a healthy, optimal alveolar ridge [1]. This survival is attributed to advancements in implant technology, surgical techniques, and patient management. For a successful implant-supported dental treatment, it is essential to have a sufficient volume and quality of alveolar bone to support the insertion of dental implants and enable the placement of fixed partial dentures or removable prosthetics. An optimal ridge not only provides mechanical stability but also supports proper osseointegration, a crucial biological process where bone integrates with the surface of the implant, forming a secure attachment [2].

Despite the advancements in dental implantology, the literature surrounding implant failure remains variable and sometimes contradictory. The rates of failure are influenced by multifactorial aspects, and the distinction between early and late failures is critical. Early implant failures, occurring prior to the prosthetic phase, have been extensively studied and are often attributed to the failure of bone tissue to establish osseointegration. A 2011 literature review emphasized this, showing that the absence of proper bone integration before prosthetic restoration is a common cause of early failures [3].

A meta-analysis that aimed to investigate risk factors associated with early implant failure provided insights into both patient-related and implant-related factors. It concluded that significant risk factors for early failures included smoking habits, implants shorter than 10 mm, and implants placed in the maxillary region [4]. These findings underscore the importance of identifying patients who might be at higher risk and adapting surgical and postsurgical protocols accordingly.

Further exploration of the predictors of implant survival or failure was conducted in a 2008 study, categorizing them into patient-related factors such as general health, smoking status, oral hygiene practices, bone quality and quantity, as well as implant location, implant-specific characteristics, and the clinician’s experience [5]. The findings highlighted that comprehensive patient assessments and preoperative planning are paramount to reduce the risk of failure.

In recent years, a 2020 study shed light on the time distribution of failures, revealing that early implant failures were approximately twice as common as late failures. Late failures, which occur after the osseointegration phase and during functional use, were frequently associated with factors such as the quality of cancellous bone in older adults [6]. This distinction emphasizes that while early failures are often linked to surgical and biological factors, late failures may involve biomechanical and systemic influences.

Other literature reviews have highlighted additional factors impacting implant survival or failure, including the accuracy of implant positioning [7], the type of prosthetic treatment [8], bone quality and quantity [9], the presence of occlusal trauma [10], and systemic health conditions such as diabetes and osteoporosis [11]. These findings demonstrate the complex interplay of biological, mechanical, and patient-related variables in implantology.

The dental field has traditionally focused on evaluating success through rates of implant failure and survival. A systematic review encompassing 783 publications found that graft survival was a prominent metric for assessing implant success, with implant survival being the most frequently reported outcome at 58.2% and often serving as the sole criterion for evaluating the survival of sinus floor elevation procedures [12]. When implants are placed in a fully healed, uncompromised alveolar ridge, they typically demonstrate high clinical success and survival rates [13]. In this comprehensive study, implant survival serves as the primary measure of success.

## 2. Materials and Methods

### 2.1. Study Design and Data Source

We conducted a retrospective cohort study utilizing the electronic health records (EHR) from “Maccabi Dent”, the dental division of Maccabi Health Services, Israel’s second-largest Health Maintenance Organization (HMO). This study encompassed patients who underwent various dental implant procedures between early 2014 and late 2022. The treatments varied in implant type, adjunctive bone grafting procedures, implant location (maxillary or mandibular), and whether sinus augmentation was performed.

### 2.2. Data Collection and Processing

Data were extracted retrospectively from the EHR system of Maccabi Dent clinics across Israel. This dataset included clinical aspects of dental implant treatments, encompassing procedures such as implants with or without concomitant bone grafting and sinus augmentation.

### 2.3. Data Extraction Process

Source and scope: The primary data source was the EHR system used by Maccabi Dent clinics, containing detailed records of all patient treatments from early 2014 through late 2022.

Treatment codes: Treatments were cataloged using standardized codes to ensure uniformity. These codes allowed for precise identification of procedures performed, including implant placement, grafting, and sinus augmentation, and enabled the stratification of data by treatment type, implant location, and adjunctive procedures.

Data anonymization: To maintain patient confidentiality, all identifying information (such as names, ID numbers, and contact details) was removed during the initial data extraction by Maccabi’s computing department. This anonymization process complied with data protection standards and ensured patient privacy before any data were transferred to the research team.

### 2.4. Data Processing and Preparation

Database construction: The anonymized data were transferred to the research team, where a study-specific database was created. This database was tailored to contain only the relevant clinical variables required for analysis, excluding extraneous data to streamline the research process.

Data cleaning and quality control: The data underwent rigorous cleaning to correct inconsistencies, such as duplicate entries or incomplete records. Quality control checks were implemented to ensure data accuracy and completeness, including cross-verification of treatment codes and consistency checks across clinical outcomes.

Filtering and refinement: Further filtering was applied to align the dataset with the study’s focus. Only records meeting specific inclusion criteria (e.g., patients with complete follow-up data) were retained in the final dataset.

### 2.5. Data Structuring for Analysis

The final dataset was structured to facilitate statistical analysis, with each patient record containing variables representing demographics, treatment type, implant location, procedure-specific details, and clinical outcomes. This structure enabled a comparative analysis of outcomes across different treatment types and locations.

### 2.6. Study Objectives and Outcome Measures

The primary objective of this study was to assess clinical outcomes and patterns of dental implant use, including implant survival across different implant types and techniques. The outcome measures included the following:

Clinical survival rate: This refers to the survival rate of implants based on follow-up records, determined by implant stability and absence of complications.

Comparative Analysis by Implant Location and Procedure Type: We evaluated differences in survival rates across mandibular vs. maxillary locations and in cases with or without adjunctive bone grafting or sinus augmentation.

Variables were categorized as independent and dependent. Independent variables were as follows: 1. implant locations (maxillary versus mandibular); 2. surgical procedure types (presence or absence of bone grafting); 3. implant number (single vs. multiple); 4. gender (male vs. female); 5. prosthesis types (fixed vs. removable); and 6. socioeconomic status (low, middle, high). In Israel, socioeconomic status is calculated using an index composed of 14 different variables in the areas of demographics, education, employment and pensions, and standard of living. This index is divided into 10 clusters. For calculation purposes, we have defined the following levels:

Low: clusters 1–3;

Medium: clusters 4–7;

High: clusters 8–10.

The dependent variable was survival vs. failure.

### 2.7. Statistical Analysis

Descriptive statistics were used to summarize baseline characteristics, with continuous variables presented as means and standard deviations and categorical variables as frequencies and percentages. To assess statistical significance, all analyses were performed using IBM SPSS Statistics software version 27.0, with a significance level set at 0.05.

### 2.8. Ethics Approval

The study protocol was reviewed and approved by the Institutional Review Boards of Maccabi Health Services (MHS-0090-20) and Assuta Hospital, Tel Aviv (ASMC-0032-20).

## 3. Results

### 3.1. Study Population and Implant Types

Between 2014 and 2022, a total of 54,210 patients (aged 18–88; 55% female, 45% male) underwent dental implant procedures at Maccabi Dent clinics, Israel. After data processing and filtering, this study included 158,824 eligible implants, with details on implant type, location, and associated rehabilitations.

- Implant scenarios: The implant procedures included bone grafting (28.8%), open sinus lifting (10.2%), closed sinus lifting (6.2%), and other unspecified procedures (54.8%). The highest failure rates were observed in closed sinus lifting procedures at 3.96% and the lowest in cases without sinus lifting or bone grafting at 2.01% (see Table 1).

### 3.2. Implant Survival and Failure Rates

**Figure 1:**
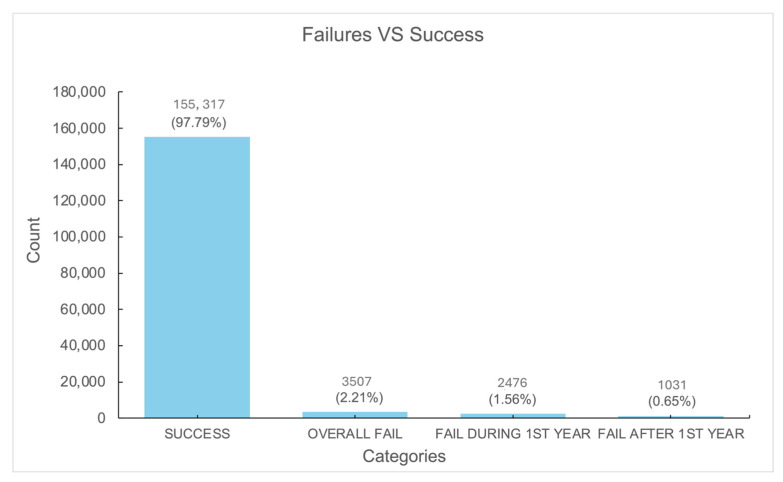
A. Survival and failure of dental implants.

Of the 158,824 implants, the overall survival rate was 97.79%, with a total failure rate of 2.21%. Failures within the first year accounted for 1.59% of cases (Figure 1).

### 3.3. Gender Differences in Implant Outcomes

**Figure 2:**
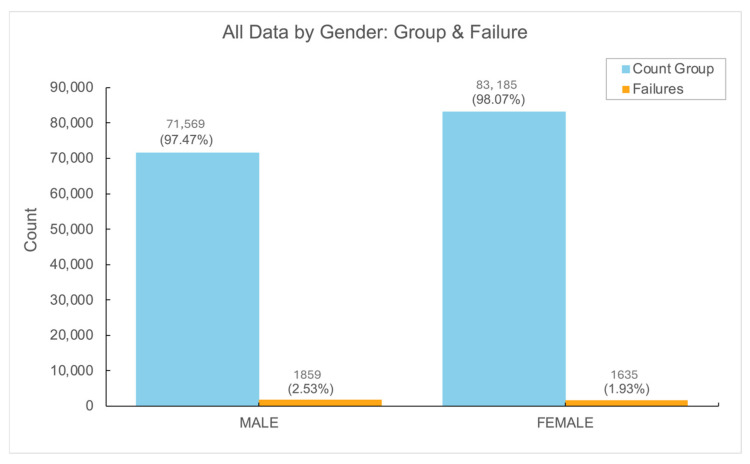
Gender differences in number of dental implants and failure rate.

Among the study population, implant failures were significantly higher in men compared to women, with the overall implant failure rate in men reaching 2.53% vs. 1.93% (*p* < 0.01). Generalized estimating equation (GEE) analysis indicated that the male gender was associated with increased relative risk of implant failure (RR 1.21, 95% CI 1.07–1.37, *p* = 0.002) (See Figure 2).

### 3.4. Socioeconomic Status and Implant Failure

Analysis across low-, middle-, and high-socioeconomic-status groups revealed minimal variation in failure rates, with differences remaining within 0.5%. No statistically significant difference was observed among socioeconomic groups (*p* = 0.70).

### 3.5. Implant Failure by Location

**Figure 3:**
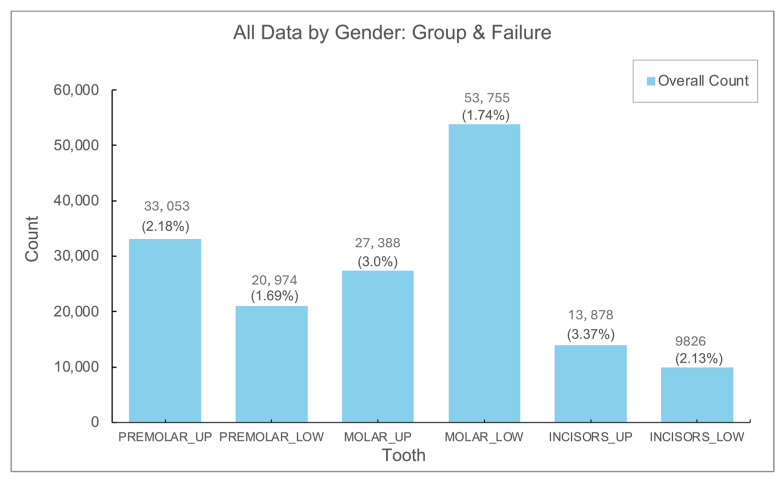
Number and rate (%) of failure in different locations.

Implants in the lower jaw, especially in the molars, represented the most frequent implant placements. The lowest failure rates were found in premolars and lower molars, whereas the highest failure rates were noted in upper jaw molars and incisors, with statistically significant differences (*p* < 0.01) (Figure 3).

### 3.6. Rehabilitation Type and Failure Rates

Permanent restorations, including crowns and fixed prostheses, accounted for 96.1% of all implant-supported restorations, with a failure rate of 3.74%. In contrast, removable restorations constituted only 3.9% of cases, but with a significantly higher failure rate of 9.32% (*p* < 0.01).

## 4. Discussion

In this study, we aimed to assess the survival and failure rates of dental implants within the context of a clinical database. Implant survival and failure were defined based on the presence or absence of the implant in its original site and its continued functionality, as outlined in previous studies [14,15]. This approach has been widely adopted in implant dentistry, where the assessment of implant survival is often tied to the clinical presence of the implant and its ability to function effectively within the mouth. In contrast, other studies, such as those conducted by Buser et al., introduce radiographic criteria to evaluate implant failure, particularly focusing on peri-implant radiolucencies as an indicator of failure [16,17]. These varying definitions underscore the importance of understanding the methodological differences in implant survival and failure assessments across studies.

Data from the Maccabi Dent clinical database, which encompasses a large cohort of patients, revealed an overall high implant survival rate and low failure rates. This is in agreement with the existing body of literature, which has generally shown favorable outcomes for dental implants, especially when appropriate patient selection and surgical techniques are employed. However, a closer examination of the failure patterns revealed some interesting trends. Failure rates were found to be highest within the first year following implantation, which aligns with the findings of several other studies [18,19] that have highlighted the initial postoperative period as critical for implant survival. It is during this early period that complications such as infection, implant mobility, or peri-implantitis are most likely to occur. After the first year, the incidence of failure dropped significantly, a trend that has been observed in many studies examining long-term implant survival [20,21].

Interestingly, our study diverged from the conventional approach to failure rate calculation, which typically involves the use of an arithmetic approach. In many studies, failure rates are calculated based on simple arithmetic means, which may not always accurately reflect the true risk of implant failure in a clinical setting. In contrast, our study employed a more nuanced analysis of failure rates over time, revealing a decline in failure rates after the initial year. This observation suggests that, once the initial postoperative period is overcome, implants are generally more stable, with fewer complications arising in subsequent years.

One of the hypotheses in implant dentistry is that performing multiple implants in a single patient increases the complexity of the procedure and, consequently, the risk of failure. Longer surgical durations are often thought to contribute to a higher risk of complications, including infection or implant failure. However, our analysis of the failure rates between single and multiple implants demonstrated that the difference was minimal. The failure rate for multiple implants was only 0.18% higher than that for single implants, a difference that was not statistically significant. This finding suggests that, despite the theoretical risk associated with longer surgeries, the risk of implant failure does not significantly increase when multiple implants are placed during a single surgical session. These results are consistent with some studies that have shown comparable survival rates for single and multiple implants, provided that proper surgical protocols and patient selection criteria are followed [22].

A particularly surprising finding in our study was the significantly higher failure rate in male patients, despite the fact that there were fewer male patients (73,428) compared to female patients (84,820). This gender-related difference in implant failure rates has not been well documented in the existing literature. While some studies have suggested that there may be gender-based differences in implant survival [23], the evidence remains sparse and inconsistent. Our study contributes to this ongoing debate by providing evidence of a statistically significant higher failure rate in male patients. Further research is needed to elucidate the underlying causes of this gender discrepancy. Potential factors include hormonal influences, differences in bone density, or even variations in oral hygiene practices and patient compliance, which could all contribute to differing implant outcomes between genders [24].

Another important aspect of implant failure is the anatomical location within the mouth where the implant is placed. The literature has consistently suggested that implant placement in the upper jaw (maxilla) is associated with higher failure rates compared to the lower jaw (mandible) [25]. This is thought to be due to factors such as reduced bone density in the maxilla, which can affect primary implant stability. Our analysis of the failure rates by jaw location confirmed this hypothesis, with implants placed in the maxilla exhibiting a failure rate approximately twice as high as those placed in the mandible. This finding is consistent with the body of research highlighting the challenges of maxillary implants, particularly in patients with insufficient bone volume or poor bone quality. These results underscore the importance of careful preoperative planning, including the use of bone grafting techniques or alternative implant placement strategies, when considering maxillary implants.

The relationship between the type of prosthesis used and implant failure has also been a topic of significant interest. Some studies suggest that removable prostheses supported by implants are more prone to failure than fixed prostheses, possibly due to factors such as the mobility of the prosthesis or the greater risk of mechanical complications [26]. In our study, we found that implants supporting removable prostheses had a failure rate up to twice as high as those supporting fixed partial prostheses. Although removable prostheses represent only a small proportion of the implant-supported restorations in our cohort (approximately 4%), this finding aligns with the literature indicating that fixed restorations tend to be more stable and durable in the long term [27]. The higher failure rates associated with removable prostheses may be attributed to factors such as improper occlusion, patient non-compliance with maintenance protocols, or the potential for loosening of components over time [28].

## 5. Conclusions

Our study contributes valuable insights into the survival and failure rates of dental implants, highlighting several factors that influence implant outcomes, including gender, anatomical location, and prosthesis type. Our findings align with the existing literature in many respects, while also providing novel insights into the role of gender and the relative risk associated with different types of restorations. Despite the relatively high survival and survival rates observed in our cohort, it is important to recognize the limitations of our study, including its retrospective nature and reliance on data from a single clinical database. Future studies, particularly those with a larger and more diverse patient population, are needed to further explore the factors influencing implant survival and failure.

## Figures and Tables

**Table 1:** The number and the rate of the different types of dental implants.

Group	Count Group (%)	Failure Rate
Dental Bone + Grafting	45,715 (28.8)	2.17%
Open Sinus Lifting	16,211 (10.2)	2.94%
Closed Sinus Lifting	9820 (6.2)	3.96%
Others	87,078 (54.8)	2.01%

## Data Availability

The original contributions presented in the study are included in the article, further inquiries can be directed to the corresponding author.

## Abbreviations

HMO	Health Maintenance Organization
GEE	generalized estimating equation
